# Evaluation of the Pulp Oxygen Saturation Reading after Tooth Bleaching: A Randomized Clinical Trial

**DOI:** 10.1155/2022/1598145

**Published:** 2022-04-28

**Authors:** Dilma Helena Neves Henriques, Ana Maria Hecke Alves, Larissa Fernanda Pottmaier, Lucas da Fonseca Roberti Garcia, Eduardo Antunes Bortoluzzi, Cleonice Silveira Teixeira

**Affiliations:** ^1^Department of Dentistry, Federal University of Santa Catarina, Florianópolis, Santa Catarina, Brazil; ^2^Department of Dentistry, ABCD Magic School, Florianópolis, Santa Catarina, Brazil; ^3^Department of Diagnosis & Oral Health, Division of Endodontics, School of Dentistry, University of Louisville, Louisville, KY, USA

## Abstract

**Objectives:**

The purpose of this clinical trial was to evaluate the influence of in-office dental bleaching on the pulp oxygen saturation (SpO_2_p) reading. *Material and Methods*. SpO_2_p was measured using a pulse oximeter in 112 upper and lower anterior teeth (canines and incisors) of patients submitted to bleaching. Whitegold Office 35% (WGO) (upper and lower left hemiarch) and Whiteness HP Auto Mixx 35% (WHP) (upper and lower right hemiarch) bleaching agents were used. SpO_2_ measurements (teeth and index finger) were taken before and after each of the three application sessions of the agents. In the 4^th^ session, in which no bleaching gel was used, only SpO_2_ was measured. Before and after the bleaching sessions, a colorimetric device performed the teeth color reading. The waiting time between sessions was 7 days. Data were analyzed by the Friedman, Kruskal–Wallis, and Mann–Whitney *U* tests (*α* = 0.05). Color change (Δ*E*) data were correlated (Spearman's Rho test) with the SpO_2_p levels.

**Results:**

Neither of the two bleaching agents showed significant differences between the readings when evaluated individually (WGO, *P*=0.780, and WHP, *P*=0.494). When taken together, the results showed significant difference between the readings performed, with higher median values after bleaching sessions: before (97.3) and after (98.6) 1^st^ session; before (98.3) and after (98.3) 2^nd^ session; before (98.3) and after (99.0) 3^rd^ session; and after 1 week (98.3). The dental groups formed by maxillary lateral incisors (*P*=0.012) and mandibular incisors (*P* < 0.001) showed a significant difference. Spearman's Rho test showed a nonsignificant and weak correlation between Δ*E* and SpO_2_p in most comparisons.

**Conclusions:**

The in-office dental bleaching influenced the SpO_2_p reading, regardless of the dental group evaluated or the bleaching agent used. *Clinical Relevance*. This study provides information about the influence of in-office tooth whitening on the pulp SpO_2_p levels. The observation of pulp vitality during and after the use of bleaching agents is important for the follow-up of patients undergoing tooth whitening. The use of a pulse oximeter may be a viable and painless alternative to perform this monitoring. The clinical trial was registered with the Brazilian Registry of Clinical Trials (ReBEC; registration number: https://clinicaltrials.gov/ct2/show/RBR-68xbth).

## 1. Introduction

The proper diagnosis of an injured pulp tissue may hinder the clinical routine of professionals, as its response to external stimuli is affected by the occurrence of inflammatory processes [1, 2]. These changes jeopardize the quality of the pulp condition diagnosis, especially when sensitivity tests, as the thermal [3] and electrical [4] types, are used for this purpose. Such tests are based on the neural response of the pulp, which, when reduced, may induce the professional to an incorrect diagnosis of pulp necrosis [2].

The ideal diagnostic method is one that assesses vitality through the supply of pulp vascularization [3–6]. Even when there is no sensory (neural) response, the pulp tissue may remain vital due to the noninterruption of blood supply [2]. Therefore, pulp necrosis cannot be determined based solely on the negative response provided by conventional sensitivity tests [6]. Conversely, technological advances have led to the development of alternative and noninvasive methods for the evaluation of pulp vascularization, such as spectrophotometry, laser Doppler flowmetry, and pulse oximetry, the latter being considered the most promising diagnostic method [6–8].

Pulse oximetry is a noninvasive physiometric method used to determine the peripheral oxygen saturation (SpO_2_) of oxygenated hemoglobin (oxyhemoglobin) in the blood [9]. In other words, SpO_2_ is the percentage of the level of total arterial oxygen saturation (SaO_2_) present in the blood (oxyhemoglobin and deoxyhemoglobin) [10, 11]. The pulse oximeter (PO) consists of a device that has a sensor with two light-emitting diodes [11]. These light emissions are captured by a photodiode receptor and transformed by electronic circuits in SpO_2_ and heart rate measurements [12]. The light-emitting diodes operate at wavelengths of 660 nm (red light) and 900–940 nm (infrared light), whose emissions, when passing through the tissues, are absorbed by oxyhemoglobin and deoxyhemoglobin [5, 13]. The extent of absorption of the two light waves, which identify oxygenated hemoglobin (arterial blood) and deoxygenated hemoglobin (venous blood), is responsible for providing the SpO_2_ levels [13, 14].

Oximetry in dentistry has been applied as a diagnostic method in several clinical situations [4, 15]. However, in order to enable its application in the pulp SpO_2_ (SpO_2_p) reading of the different dental groups [15, 16], the oximeter sensors have been adapted according to the anatomy of the tooth to be analyzed, such as incisors and canines [5], lower premolars [15], upper premolars [8], and upper and lower molars [17].

Few clinical studies have evaluated the SpO_2_p levels in both sound deciduous and permanent teeth, indicating the use of oximeter sensors for the diagnosis of pulp vitality [5, 18] and evaluation of pulp inflammation [6]. The literature also suggests a possible correlation of lower SpO_2_p rates found in anterior permanent teeth with periodontal diseases [19]. Pulse oximetry proved to be more reliable than thermal and electrical tests in assessing the vitality of teeth with a history of recent trauma, over a period of 6 months [1].

Findings in the literature suggest that in-office dental bleaching causes changes in the normal pulp tissue response, such as the short-term occurrence of greater tooth sensitivity [20]. Studies have shown that the upper central incisors had a temporary decrease in SpO_2_p levels after in-office and/or at-home tooth bleaching [21, 22]. However, SpO_2_p levels returned to the normal levels at the end of the bleaching procedure [21, 22]. Injuries to the pulp tissue caused by tooth bleaching may be related to the hydrogen peroxide concentration of the bleaching agent and the time it remains in contact with the tooth [23, 24]. Moreover, some of these injuries may even promote pulp necrosis of the tooth submitted to bleaching [23]. Despite the existence of studies on the subject, the findings reported in the literature, evaluating supposed changes in SpO_2_p levels in teeth submitted to bleaching, are still insufficient.

Therefore, it is crucial that *in vivo* studies must be performed to assess possible changes in SpO_2_p levels resulting from tooth bleaching. The purpose of this clinical trial was to evaluate the PO reading ability of SpO_2_p levels before and after in-office dental bleaching. The null hypothesis tested was that the in-office dental bleaching would not interfere with the reading of the oxygen saturation levels of the pulp tissue, regardless of the dental group evaluated, the bleaching agent used, or the number of bleaching sessions.

## 2. Methodology

The clinical trial was registered with the Brazilian Registry of Clinical Trials (ReBEC; registration number: RBR-68xbth). It was carried out in accordance with the ethical standards laid down in the 1964 Declaration of Helsinki and its later amendments and approved by the Human Research Ethics Committee of the Federal University of Santa Catarina (reference no. 73198217.1). This study followed the Consolidated Standards of Reporting Trials (CONSORT) guidelines.

### 2.1. Sample Size Calculation

The sample size was estimated based on studies that evaluated SaO_2_p in 67 anterior teeth [18], 17 upper incisors [1], and 80 canines and premolars [25]. Therefore, for analysis with *α* = 0.05 and 80% power, a minimum number of 15 teeth per group was initially defined, and the sample size was estimated at 105 teeth. The patients included in the study were asked to sign the informed consent form, necessary for the continuation of the research.

### 2.2. Study Design

This study was a randomized double-blind (participant and operator), single-arm, single-group clinical treatment trial performed between August 2017 and June 2019. The same distribution of the number of teeth per group was performed, as well as the design of the experiment, according to the CONSORT guidelines. The study was submitted and approved by the Brazilian Registry of Clinical Trials (Protocol No. RBR-68xbth).

### 2.3. Selection of Patients

Patients undergoing tooth-bleaching procedures at the Dental Clinics of the Federal University of Santa Catarina (UFSC), who already had periapical radiographs, were invited to participate in the research. The study excluded smoking patients, patients who had previously undergone external dental bleaching, patients with parafunctional habits, patients with a history of dental trauma, pregnant women, lactating women, patients using orthodontic appliances, and those who reported previous spontaneous tooth pain. The study included healthy volunteers of both genders' nonsmokers, sound permanent teeth without cavities or restorations, teeth with pulp vitality without signs of cracks or internal resorption or calcification, and a complete root apex without signs of periodontal or periapical lesions. Initially, 17 patients were selected (8 males and 9 females), aged between 18 and 24 years (mean age of 22.3 years), with good health in general (ASA I), and who met the eligibility criteria. However, one of the patients gave up on the procedure before it started. Thus, 16 patients continued in the study, in which a total of 136 teeth were selected (82 upper and 54 lower teeth).

### 2.4. Intervention

#### 2.4.1. Bleaching Procedure

Prior to the initial color evaluation, prophylaxis was performed with a Robinson brush and prophylactic paste on all patients' teeth in order to promote the elimination of extrinsic stains. The prophylaxis was repeated before each bleaching session. Then, relative isolation of the teeth was performed with a lip retractor and the use of a gingival barrier (Top Dam; FGM, Joinville, SC, Brazil), which covered the marginal gingiva and papillae in order to protect them. Two different bleaching agents, Whiteness HP Auto Mixx 35% (FGM, Joinville, SC, Brazil) and Whitegold Office 35% (Dentsply, São Paulo, SP, Brazil) were evaluated for their effect on the SpO_2_p readings. The patients were not aware of the dynamics adopted during the application of the bleaching agents (in which hemiarches would be used) and the data resulting from the SpO_2_p reading.

The bleaching agents' application was carried out as follows: G1—the Whiteness HP Auto Mixx 35% (WHP) gel was applied to the upper and/or lower right hemiarches—and G2—the Whitegold Office 35% (WGO) was applied to the upper and/or lower left hemiarches. The bleaching agents remained in contact with the buccal teeth surface for 45 minutes. Then, they were gently removed with a gauze and flushed with water. In total, the patients received three applications of the bleaching agents, once per week. SpO_2_ measurements (teeth and index finger, mean of three measurements for each of them) were taken before and after each of the three application sessions of the agents. In the 4^th^ session, in which no bleaching gel was used, only SpO_2_ was measured. The waiting time between sessions was 7 days. The in-office dental bleaching procedures were performed by a specialist in endodontics with the aid and collaboration of a specialist in restorative dentistry. In addition, the readings of SpO_2_ levels were taken by the same operator specialized in endodontics.

The color measurements of the teeth were performed according to the CIELAB system (*L*^*∗*^ coordinate represents the lightness; *a*^*∗*^ coordinate represents the color and saturation of the green-red axis; and *b*^*∗*^ coordinate represents the color and saturation of the blue-yellow axis). A colorimetric device (VITA Easyshade Advance 4.0; VITA Zahnfabrik, Bad Säckingen, Germany) was used as a quantitative evaluation method to perform the color readings. An initial color measurement was performed at the center of the buccal surface of each tooth. New color measurements were performed after each bleaching session (first, second, and third, and 1 week after the third session). The coordinates *L*^*∗*^*a*^*∗*^*b*^*∗*^ were recorded, and the mean values were calculated. The data of the initial color measurement of the coordinates *L*^*∗*^*a*^*∗*^*b*^*∗*^ were used as reference values (baseline). The color change (Δ*E*) before and after each bleaching session was calculated using the formula “Δ*E* = [(Δ*L*^*∗*^)2 + (Δ*a*)2 + (Δ*b*)2]1/2,” where Δ*L*, Δ*a*, and Δ*b* represent the differences in lightness, green-red axis, and blue-yellow axis, respectively. In order to ensure that only the central area of the buccal surface of each tooth was measured, a condensation silicone matrix (Zetaplus; Zhermack, Badia Polesine, Italy) was fabricated and adapted at the tip of the colorimetric device during its use. The color measurements were performed in triplicate for each tooth, under standard illumination, by the same operator.

#### 2.4.2. SaO_2_p Reading on Teeth Using a PO

Randomization of SpO_2_p level readings was performed by randomly selecting the arcade measured initially with the aid of a coin. The first time the coin was launched, the obverse side corresponded to the upper arch and the reverse side to the lower arch. After selecting the arcade, the coin was launched a second time, to then select in which hemiarch the reading would start from, where the obverse side corresponded to the right hemiarch and the reverse side to the left hemiarch. If the upper left hemiarch was drawn first, after its conclusion, the SpO_2_p level reading continued with the upper right hemiarch. Next, the coin was launched again to select in which lower hemiarch the reading should continue. If the lower left hemiarch was drawn, then the last reading would be that of the right lower hemiarch. The dynamics of the draw was performed in each one of the four bleaching sessions, as described above.

In order to perform the SpO_2_p level reading, before and after the dental bleaching sessions, the PO Sense! 10 (Alfamed Sistemas Médicos Ltda, Brazil) was used, associated with the 3025 sensor (Smiths Medical PM Inc., Waukesha, WI, USA) for teeth positioning. To ensure proper adaptation to the tooth at the time of each reading, the sensor was attached to a stainless-steel support specially designed and custom-made for this purpose (5).

During PO measurements, in addition to the use of a retractor for lips, mucosa, and tongue, and the isolation of the marginal gingiva and papillae with the use of a gingival barrier, a transparent PVC film covered the teeth, which avoided the direct contact of the PO sensors with saliva and oral tissues. Furthermore, other precautions were taken, such as orienting the patient about keeping their head completely still; keeping the reflector light off; standardization of the waiting time in 30 seconds for reading of the PO after triggering; and switching off the device for 1 minute between each reading performed. The stabilization of the sensor was guaranteed by a stainless-steel positioner in order to maintain parallelism and embrace the coronal surfaces of the teeth (and allow the light emitted by the emitting sensor to reach the receiving sensor without gingival tissue interference).

**Figure 1:**
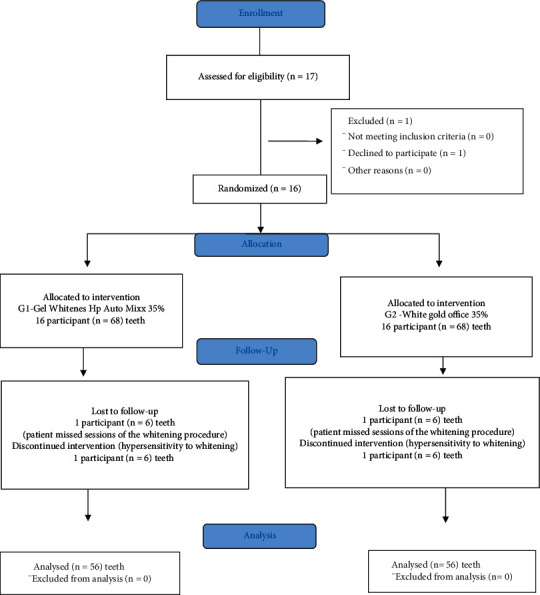
Flow diagram of study inclusion criteria.

Of the 16 patients who started the in-office dental bleaching, two withdrew during the experiment and were excluded from the study. The former because he reported hypersensitivity after the first bleaching session, and the latter was unable to attend all sessions on the scheduled dates (Figure 1). Thus, 14 patients continued in the study; of whom, a total of 112 teeth were evaluated, 70 upper and 42 lower teeth (Table 1).

#### 2.4.3. Statistical Analysis

Statistical analysis was performed using SPSS 21.0 for Windows (SPSS Inc., Chicago, IL, EUA). The level of significance was set at 5%. The collected data did not show normal distribution (Shapiro–Wilk, *P* < 0.05). Therefore, the statistical analysis was performed using the nonparametric statistical tests of Friedman, Kruskal–Wallis, and Mann–Whitney *U*. Additionally, the color change (Δ*E*) data were correlated (Spearman's Rho test) with the SpO_2_p levels measured at the different periods (after the first, second, and third bleaching sessions and at the final, that is, one week after the third session).

## 3. Results

The results are shown in Tables 2–5.  Table 2 shows the results of the comparison of SpO_2_p levels among the different periods of reading, before and after the use of the bleaching agents (WGO × WHP), irrespective of the dental group, and also the comparison with SpO_2_ values measured on the patient's finger.

When the bleaching agents were individually evaluated, no significant difference was found (Friedman, *P* > 0.05) among the values recorded over the different periods of reading for both WGO (*P*=0.780) and WHP (*P*=0.494). When compared with each other, there was a significant difference (Mann–Whitney *U*) between the bleaching agents only in the evaluation performed after the 3^rd^ bleaching session (*P*=0.044). No significant differences were found between the agents used in the other periods of reading (*P* > 0.05) and for SpO_2_ levels measured using the PO on the patient's finger (control group, *P*=1.00).

Table 3 shows the results of the comparison among the levels of oxygen saturation over the different periods of reading as a whole, with no distinction between the dental groups and bleaching agents. The Friedman test found a significant difference among the values recorded over the different periods of reading (*P* < 0.0001), with higher values of oxygen saturation after using the bleaching agents.

Table 4 shows the SpO_2_p results achieved at the different periods of reading and irrespective of the bleaching agents used on the teeth: upper central incisor (UCI); upper lateral incisor (ULI); lower central incisor + lower lateral incisor (LCI + LLI); and upper canine + lower canine (UC + LC). A significant difference (Friedman, *P* < 0.05) was found only in the dental groups ULI (*P*=0.012) and LCI + LLI (*P* < 0.001). The values of the dental groups UCI (*P*=0.074) and UC + LC (*P*=0.706) did not show a significant difference over time. A significant difference was found (Kruskal–Wallis) after the 1^st^ bleaching session (*P*=0.016), after the 2^nd^ session (*P* < 0.001), after the 3^rd^ session (*P*=0.02), and final reading (*P*=0.009). The comparison between SpO_2_p levels and SpO_2_ levels on the fingers of each patient showed no significant difference (Mann–Whitney, *P*=0.328).

The results obtained after Spearman's Rho correlation test are shown in Table 5. Nonsignificant and weak correlations (*P* > 0.05) were observed in most comparisons. When the correlations were significant (SpO_2_p after 1^st^ × Δ*E*2 = 0.406, and SpO_2_p after 1^st^ × Δ*E* Final = 0.410), they were linear, positive, and moderate.

## 4. Discussion

The purpose of this study was to evaluate *in vivo* the influence of in-office dental bleaching on the SpO_2_p reading in various dental groups (incisors and canines). When the two bleaching agents were evaluated together, a higher SpO_2_p level reading after the bleaching sessions was observed. The different dental groups also influenced the readings performed, with significant differences for the upper lateral incisors and lower incisors. Based on these results, the null hypothesis that “the tooth bleaching would not interfere with the reading of the SpO_2_ levels of the pulp tissue, regardless of the dental group evaluated, the bleaching agent used, or the number of bleaching sessions has to be rejected.”

For the in-office dental bleaching, two different agents were used, Whiteness HP Auto Mixx 35% (WHP) and Whitegold Office 35% (WGO). Both bleaching agents contain 35% hydrogen peroxide in their composition. Despite the efficiency in dental whitening, bleaching agents contain chemical substances that, if they reach the pulp tissue, may affect pulp vitality, promoting several inflammatory processes, such as pulpitis, followed by an eventual pulp necrosis [23, 26]. WHP gel, unlike WGO gel, contains soluble calcium in its composition, which, according to some studies, causes less sensitivity [27] and contributes significantly to minimize the decrease of enamel microhardness [28].

The two bleaching agents were used in the bleaching sessions and compared with each other taking into consideration their effect on the SaO_2_p reading using the PO. Neither of the agents, when evaluated individually, showed significant differences between the several readings performed (WGO *P*=0.780 and WHP *P*=0.494). However, as previously mentioned, when the bleaching agents were evaluated simultaneously, a significant difference was observed between the readings performed, with higher values after the bleaching sessions. The completion of dental bleaching promotes the release of free radicals from the dental structure by the hydrogen peroxide action, which by breaking the double bonds of the pigment molecules allows a greater number of simple bonds between the carbon chains, with a clearer structural effect [29]. Hence, it is understood that bleaching promotes a greater passage of light by the dental structure [29], which may have, enabled higher values in the reading of the devices.

Studies have reported that the PO presents greater sensitivity and specificity in the evaluation of pulp vitality, when compared with thermal and electrical tests [15, 25, 30]. The oximeter chosen for this study was Sense! 10, which is more sensitive in cases of low perfusion (poor, low, or unsatisfactory perfusion). This device allows SpO_2_ readings between 0% and 100%, and a pulse between 25 and 300 bpm (information provided by the manufacturer). Niklas et al. [31] have shown that the sensitivity of the oximeter varies for different wavelengths of light emission from the device used. Starting at a minimum of 20% for 400 nm values, the sensitivity increases almost linearly up to 100% for the 850 nm range and then decreases again. At 950 nm, as well as in the 650 nm range, the sensitivity is reduced to approximately 80% [31]. Also, the reading of the device is more affected in the range of 850 nm by disturbances due to interference from gingival tissue, among others.

On the contrary to what was observed in other studies [5, 8, 32], there was no significant difference between the SpO_2_ values obtained on the patient's finger and the SpO_2_p values. Perhaps, this result might be explained by the use of different models of oximeters in other studies, with less sensitivity than that used in the present study (Sense! 10). In addition, to operate in conjunction with the PO Sense! 10, we opted for the 3025 sensor coupled to a custom-made support [5]. This support ensures the parallelism of the light-emitting and receiving diodes, as well as better adaptation of the sensor to the crowns of deciduous and permanent anterior teeth, in addition to preventing the light beam from reaching the gingival tissue, which would result in false readings [5, 8].

The SpO_2_p readings performed by the PO on the anterior teeth had higher median values than those already published in the literature [7]. Studies have shown lower values in SpO_2_p readings than the reading performed on the patient's finger, with average SpO_2_p levels of 87.73% for CIs, 87.24% for LIs, and 87.26% for canines [7]. In another study, the SpO_2_p mean values were 85.11% for CIs, 80.21% for LIs, and 89.55% for canines [33]. Such results have been explained first by the location of the pulp tissue, surrounded by a hard tissue, which creates an obstacle for the detection of vascularization [5, 7], and then by the diffraction of infrared light through the enamel prisms and dentinal tubules, which may lead to erroneous readings of oxygen saturation [34, 35]. The difference between the results found in our study and those presented in the literature may be attributed to several factors, such as the aforementioned greater sensitivity of the device used, as well as the very young age group (between 18 and 24 years old) of patients undergoing bleaching [33].

The dental pulp of older patients had a significant decrease in nerve bundles, a decrease in pulp volume and some changes in interstitial tissues, which may cause false responses to pulp sensitivity and vitality tests [4]. The average of SpO_2_p levels found in premolars decreased significantly in the age group of 40–44 (80%), when compared with the age groups of 20–24 (89.71%), 25–29 (87.67%), 30–34 (88.71%), and 35–39 years (84.80%) [8], despite these values were obtained from pulp tissues of different dental groups. Although there are few studies evaluating the SpO_2_p of premolars [6, 8] and molars with normal pulps (16), results of different mean SpO_2_p levels are found in the literature, according to dental groups, suggesting that the dental group may affect SpO_2_p readings [5, 8, 18, 32].

In the present study, we decided on evaluating the SpO_2_p reading in different dental groups (upper and lower anterior teeth) during different moments of in-office dental bleaching. The values recorded during the evaluations showed a significant difference for the upper lateral incisor and lower incisor groups. However, in the other groups (UCI and canines), there were no significant differences in the readings performed over time. Our findings differ from the results of two recently published studies, which assessed the SpO_2_p levels in UCIs after in-office and at-home dental bleaching [21, 22]. In these studies, there was a decrease in SpO_2_p levels right after the in-office bleaching, and a significant increase in SpO_2_p levels 30 days after bleaching, when compared with the levels recorded one week [22] and right before [21] the first bleaching procedure. The controversial results may be explained by the differences in the design of the studies already published with the present study, such as the sensitivity of the PO device and the type of sensor used: the sample size, the bleaching procedure, and the bleaching agents used.

It is also pertinent to emphasize that, unlike these two studies [21, 22], our methodology did not cover at-home bleaching. Therefore, our results are based solely on the SpO_2_p readings obtained before and shortly after the in-office bleaching sessions. This fact may justify not having noticed a significant reduction in the values of the SpO_2_p reading during the sessions. There was a short period between the bleaching agents' application and the SpO_2_p readings, which might have been insufficient for the installation of an inflammatory process [36]. On the other hand, there was a period of 7 days between SpO_2_p readings from one session to another, which may have been enough for the inflammatory process to cease. Corroborating this hypothesis, Silva-Costa et al. [36] have reported that bleaching caused moderate inflammation to the pulp tissue of rats' incisor at the 24-hour period, with characteristics of tissue repair after 10 days. It should also be noted that the operation of the PO is not properly understood in cases where the pulp has an inflammatory process installed, justifying further studies for this purpose [6, 7].

Spearman's test showed, in most comparisons, a nonsignificant and weak correlation between dental pulp oxygen saturation and tooth color change (Δ*E*) after the bleaching sessions. In addition, when the correlation was significant, it was linear, positive, and moderate. It is valid to notice that no other studies so far have performed such analysis, which limits the discussion of the results obtained in the present study.

Although the scientific literature shows limitations of the PO use regarding its effectiveness, which may be hindered due to patient movement, electrical interference, and low peripheral perfusion, the PO technology has been making significant advances [37]. Thus, new PO devices are more stable and are more effective in evaluating SpO_2_ in patients with low perfusion [37]. Therefore, it may be assumed that such devices, due to their greater sensitivity in the detection of SpO_2_, present a lower risk of false-negative responses in the evaluation of pulp vitality, as observed in the higher SpO_2_p values obtained in our study, when compared with other studies previously performed [5, 7, 8, 22].

## 5. Conclusion

Our results demonstrated that PO might be used as an effective method for monitoring pulp health of teeth submitted to in-office bleaching. The in-office dental bleaching influenced the pulp tissue SpO_2_ reading by the PO, regardless of the dental group evaluated or the bleaching agent used.

## Figures and Tables

**Table 1:** Distribution of the teeth that were submitted to *in vivo* SpO_2_p reading using the PO, regardless of the arch (upper or lower) and the dental groups evaluated^*∗*^.

Arch/teeth	CI	LI	C	Total
Maxillary (14 patients)	28	28	14	70
Mandibular (7 patients)	14	14	14	42
Total	42	42	28	112

^*∗*^CI: central incisor; LI: lateral incisor; C: canine.

**Table 2:** Medians (first/third quartiles) of the oxygen saturation levels measured at different periods of reading (before and after the *first*, *second*, and *third* bleaching session and at the *final*, that is, 1 week after the third session) for the different whiteners used: Whitegold Office 35% (WGO) and Whiteness HP Auto Mixx 35% (WHP)^*∗*^.

Groups (*n* = 56) 14 patients	Finger^ϯ^	Before 1^st^	After 1^st^	Before 2^nd^	After 2^nd^	Before 3^rd^	After 3^rd^	Final
WGO	98 (97/98)^aA^	97.3 (96.9/97.9)^aA^	97.8 (97.1/98.8)^aA^	98 (97.2/98.5)^aA^	97.6 (96/98.3)^aA^	97.5 (96.6/99)^aA^	98 (98/99.2)^aA^	98.1 (97.2/99)^aA^
WHP	98 (97/98)^aA^	98 (96.5/98.8)^aA^	97.6 (96.6/98.3)^aA^	98.1 (97.6/98.7)^aA^	98.1 (97.1/98.6)^aA^	98.1 (97.6/99.3)^aA^	97 (96/98.2)^aB^	97 (96.9/98) ^aA^

^*∗*^Medians accompanied by the same superscript lowercase letters in the same line did not show a significant difference (Friedman, *P* > 0.05). Medians accompanied by the same superscript capitalized letters in the same column did not show a significant difference (Mann–Whitney *U*, *P* > 0.05). ^ϯ^SpO_2_ values measured on the patient's finger.

**Table 3:** Medians (first/third quartiles) of oxygen saturation levels measured at different periods of reading, regardless of the whitening gel used or the dental group evaluated (*n* = 112, 14 patients)^*∗*^.

Before 1^st^	After 1^st^	Before 2^nd^	After 2^nd^	Before 3^rd^	After 3^rd^	Final
97.3 (96.3/98.6)^a^	98.6 (97.6/99.3)^b^	98.3 (97.3/99.2)^b^	98.3 (97.3/99)^ab^	98.3 (97.6/99.3)^b^	99 (97/100)^b^	98.3 (97.3/99)^ab^

^*∗*^Medians accompanied by the same superscript lowercase letters in the same line did not show a significant difference (Friedman, *P* > 0.05).

**Table 4:** Medians (first/third quartiles) of oxygen saturation levels measured at different periods of reading for the different dental groups^*∗*^.

Evaluation	UCI (*n* = 28) 14 patients	ULI (*n* = 28) 14 patients	LCI + LLI (*n* = 28) 7 patients	UC + LC (*n* = 28) 7 patients
Before the 1^st^ session	97.6 (95.6/98.6)^aA^	97.5 (96.3/98.9)^aA^	97 (96/98)^aA^	97.5 (96.7/98.6)^aA^
After the 1^st^ session	98.3 (97.5/99)^abA^	98.8 (98.3/99.6)^bAB^	98.6 (98/99.6)^abC^	97.8 (96.6/98.6)^aA^
Before the 2^nd^ session	98.1 (97/99.3)^aA^	98.3 (98/99.3)^aAB^	98.3 (97.1/99.2)^aBC^	98.1 (97.3/98.6)^aA^
After the 2^nd^ session	98.5 (97.7/99.6)^bcA^	98.6 (98.1/99.6)^cAB^	97.6 (96.6/98.3)^aAB^	98 (96.7/98.6)^abA^
Before the 3^rd^ session	98.6 (98/99.3)^aA^	99 (97.7/99.6)^aAB^	98.3 (97.3/99.2)^aABC^	97.8 (97.3/99)^aA^
After the 3^rd^ session	99 (98/100)^abA^	99 (99/100)^bB^	98.5 (97/100)^abcBC^	98 (97/99)^aA^
Final	98.6 (97.5/99.3)^abA^	99 (98/99.3)^bAB^	98 (97.1/99)^abBC^	97.8 (97/98.3)^aA^

^*∗*^Medians accompanied by the same superscript lowercase letters in the same line did not show a significant difference (Kruskal–Wallis and adjusted multiple comparisons, *P* > 0.05). Medians accompanied by the same superscript capitalized letters in the same column did not show a significant difference (Friedman and adjusted multiple comparisons, *P* > 0.05. UCI: upper central incisor; ULI: upper lateral incisor; LCI: lower central incisor; LLI: lower lateral incisor; UC: upper canine; LC: lower canine.

**Table 5:** The Spearman correlation value for SpO_2_p data and color change (Δ*E*) data measured at different periods.

SaO_2_p reading median (first/third quartiles)	Correlation (Spearman's Rho test)	Color change (Δ*E*)^*∗∗*^ median (first/third quartiles)			
		Δ*E*1 3.42 (2.90/4.43)	Δ*E*2 4.75 (3.89/6.54)	Δ*E*3 5.72 (4.85/6.94)	Δ*E* Final 5.67 (4.54/6.91)
After 1^st^ 98.33 (96.33/99.17)	Correlation coefficient Sig. (2-tailed)	0.334	0.406^*∗*^	0.281	0.410^*∗*^
		0.111	0.049	0184	0.047
After 2^nd^ 99.33 (98.33/99.67)	Correlation coefficient Sig. (2-tailed)	0.346	0.299	0.345	0.332
		0.098	0.156	0.099	0.113
After 3^rd^ 99.00 (99.0/100)	Correlation coefficient Sig. (2-tailed)	−0.020	−0.010	−0.052	0.049
		0.926	0.963	0.810	0.818
Final 99.00 (98.33/99.33)	Correlation coefficient Sig. (2-tailed)	−0.200	−0.117	−0.285	−0.140
		0.348	0.586	0.177	0.514

^*∗*^The correlation is significant at the 0.05 level (2-tailed). ^*∗∗*^Δ*E* means the change in teeth color value after each bleaching session (Δ*E*1: after 1^st^; Δ*E*2: after 2^nd^; Δ*E*3: after 3^rd^; and Δ*E* Final: 1 week after the third bleaching session).

## Data Availability

This study was submitted and approved by the Brazilian Registry of Clinical Trials (REBEC, Protocol No. RBR-68xbth).
